# *Clostridium difficile* ribotypes in Austria: a multicenter, hospital-based survey

**DOI:** 10.1007/s00508-015-0808-5

**Published:** 2015-07-09

**Authors:** Alexander Indra, Daniela Schmid, Steliana Huhulescu, Erica Simons, Markus Hell, Karl Stickler, Franz Allerberger, on behalf of the Austrian C. difficile Study Group

**Affiliations:** 1 Institute for Medical Microbiology and Hygiene, National Clostridium difficile Reference Laboratory, Austrian Agency for Health and Food Safety (AGES), Waehringerstr. 25a, 1090 Vienna, Austria; 2 Department of Hospital Epidemiology and Infection Control, Paracelsus Medical University, Muellner Hauptst. 48, 5020 Salzburg, Austria; 3 Administrative Department of Hospital Hygiene, Kaiser-Franz-Josef-Hospital, Kundratstr. 3, 1100 Vienna, Austria

**Keywords:** Ribotype, Moxifloxacin, Resistance, Nosocomial, Mortality

## Abstract

A prospective, noninterventional survey was conducted among *Clostridium difficile* positive patients identified in the time period of July until October 2012 in 18 hospitals distributed across all nine Austrian provinces. Participating hospitals were asked to send stool samples or isolates from ten successive patients with *C.difficile* infection to the National *Clostridium difficile* Reference Laboratory at the Austrian Agency for Health and Food Safety for PCR-ribotyping and in vitro susceptibility testing. A total of 171 eligible patients were identified, including 73 patients with toxin-positive stool specimens and 98 patients from which *C. difficile* isolates were provided. Of the 159 patients with known age, 127 (74.3 %) were 65 years or older, the median age was 76 years (range: 9–97 years), and the male to female ratio 2.2. Among these patients, 73 % had health care-associated and 20 % community-acquired *C. difficile* infection (indeterminable 7 %). The all-cause, 30-day mortality was 8.8 % (15/171). Stool samples yielded 46 different PCR-ribotypes, of which ribotypes 027 (20 %), 014 (15.8 %), 053 (10.5 %), 078 (5.3 %), and 002 (4.7 %) were the five most prevalent. Ribotype 027 was found only in the provinces Vienna, Burgenland, and Lower Austria. Severe outcome of *C. difficile* infection was found to be associated with ribotype 053 (prevalence ratio: 3.04; 95 % CI: 1.24, 7.44), not with the so-called hypervirulent ribotypes 027 and 078. All 027 and 053 isolates exhibited in vitro resistance against moxifloxacin. Fluoroquinolone use in the health care setting must be considered as a factor favoring the spread of these fluoroquinolone resistant *C. difficile* clones.

## Introduction

*Clostridium difficile* is a major identifiable infectious cause of nosocomial diarrhea [1, 2]. Clinical manifestations of *C. difficile* infection (CDI) range from asymptomatic carriage to diarrhea, pseudomembranous colitis, toxic megacolon, and death. In recent years, an increase of CDI has been reported, in part due to the spread of one specific clone, polymerase chain reaction (PCR) ribotype 027 [3–6]. PCR ribotype 078, which has been associated with both food of animals and humans, is an additional emerging strain of *C. difficile* in Europe and the USA [7, 11]. Little is known about the current dominant ribotypes of *C. difficile* among the hospitalized patients in Austria [1].

The primary objective of our study was to ascertain the frequency distribution of *C. difficile* ribotypes among *C. difficile* positive patients hospitalized in Austrian hospitals in 2012. The secondary objective was to investigate the association between the most prevalent ribotypes found and the setting *of C. difficile* acquisition (health care setting or community), the resistotypes, and the severity of CDI.

## Patients, materials and methods

### Study design and data collection

A prospective, non-interventional, descriptive, and analytical survey was conducted on *C. difficile* positive patients identified within a 4-month period, from July until October 2012 in 18 hospitals distributed across all nine Austrian provinces. The laboratories of these participating hospitals (one hospital per province, aside from Vienna, where ten hospitals participated) were asked to send either *C. difficile* toxin(s) positive specimens or *C. difficile* isolates obtained from ten successive *C. difficile* positive patients to the National *Clostridium difficile* Reference Laboratory at the Austrian Agency for Health and Food Safety (AGES). By using a standardized questionnaire, infection control officers collected information by reviewing study patients’ medical charts on demographics (sex, age), clinical signs (diarrhea defined by using Bristol stool chart typing, toxic megacolon, pseudomembranous colitis defined by gastro-intestinal endoscopy or computed tomography), epidemiological case classification (health care-associated (HA), indeterminable, and community-acquired (CA)), and on CDI-severity defined by need of surgical intervention, intensive care, or all-cause 30-day mortality. In case of hospital stay less than 30 days, information on all-cause 30-day mortality was ascertained by postdischarge interviews with the study patient or their relatives. In case of no postdischarge contact, hospital admission registers were consulted regarding readmission of the study patient within 30 days following previous admission to the study hospital [12]. According to the case definition for the mandatory *C. difficile* enhanced surveillance scheme in UK, any of the following defined a *C. difficile* infection case in patients: diarrheal stools *or t*oxic megacolon, from which a specimen tested positive for toxigenic *C. difficile* [4].

Our survey was classified as an “active surveillance study” and therefore did not require ethical approval. None of the test results was used to alter individual patient care. All patient demographic data collected were anonymous.

### Laboratory investigation

In case of toxin positive stool specimens, toxigenic culture was performed by spreading specimens on *C. difficile* agar (CLO agar containing cycloserine 0.1 g/l, cefoxitin 0.008 g/l, and amphotericin B 0.002 g/l; bioMérieux, Marcy l’Etoile, France) and incubation at 35 ± 2 °C in an anaerobic atmosphere (< 1 % oxygen, ≥ 13 % carbon dioxide), in anaerobic jars for 48 h [13]. Simultaneously the stool specimens were enriched in thioglycollate broth with vitamin K (0.5 mg/l) and hemin (5 mg/l) (Heipha Dr. Müller GmbH, Eppelheim, Germany) and incubated anaerobically at 35 ± 2 °C for 2–7 days. One 20 µl-portion, taken from the broth near the bottom of the tube, was plated directly onto *C. difficile* selective agar (CLO; bioMérieux) and incubated for 48 h anaerobically as described above. Putative *C. difficile* colonies were confirmed by testing for the common antigen (*C. difficile* agglutination test kit; Microgen, Camberley, UK) and by mass spectrometry (matrix assisted laser desorption/ionization-time of flight (MALDI-TOF) Biotyper; Bruker Daltonics, Bremen, Germany). Toxin production of strains isolated on CLO- or Columbia blood-agar (both from bioMérieux) was tested using a Toxin A + B ELFA (enzyme linked fluorescent assay) test (Vidas, bioMérieux). Ribotyping was performed as described elsewhere [14]. In vitro susceptibility testing was performed as described previously [15]. Briefly, agar-diffusion testing was performed on Brucella agar plates supplemented with hemin (5 µg/ml), vitamin K1 (1 µg/ml), and lysed sheep blood (5 % v/v) using epsilometer test (Etest®) (AB-Biodisk, Solna, Schweden) and the respective European Committee on Antimicrobial Susceptibility Testing (EUCAST) minimal inhibitory concentration (MIC) breakpoints for metronidazole and vancomycin, Clinical and Laboratory Standards Institute (CLSI) MIC breakpoints for clindamycin and moxifloxacin, and the suggested MIC breakpoints for rifampicin and an in-house disc (40 µg) diffusion test for rifaximin, as described by Huhulescu et al. [15].

*Bacteroides fragilis* ATCC 25285 and *C. difficile* ATCC 700057 were used as quality control strains. In order to investigate associations between ribotype and *C. difficile* in vitro susceptibility to metronidazole, vancomycin, clindamycin, moxifloxacin, and rifampicin, resistotypes were defined as presented in Table 1. For analyses, reduced antimicrobial susceptibility (i.e., intermediate) was considered as resistant.

**Table 1:** Definition of the resistotypes based on the results of in vitro susceptibility testing for metronidazole (metro), vancomycin (vanco), clindamycin (clinda), moxifloxacin (moxi), and rifampicin (rifam)

Resistotypes	Antibiotics				
	Metro	Vanco	Clinda	Moxi	Rifam
Resistotype 0	S	S	S	S	S
Resistotype 1	S	S	R	S	S
Resistotype 2	S	S	S	R	S
Resistotype 3	S	S	S	S	R
Resistotype 4	S	S	R	R	S
Resistotype 5	S	S	R	S	R
Resistotype 6	S	S	S	R	R
Resistotype 7	S	S	R	R	R

### Statistical analyses

Single proportion estimates were provided with 95 % confidence interval. Prevalence ratios (PRs) and 95 % confidence intervals (CIs) are presented for associations between the five most prevalent ribotypes (considered as single explanatory variables) and the outcome variables comprising the setting of *C. difficile* acquisition (health care setting or community, as defined below) resistotype (0, 1, 2, 3, 4, 5, 6, 7, defined as presented in Table 1), and the severity of disease defined as all-cause death within 30 days following *C. difficile* detection or requiring intensive care or surgical intervention.

A *C. difficile* infection case in a patient, who had symptom onset prior to or within 72 h after hospital admission, was classified as health care facility-associated (HCFA), if the patient had been discharged from a healthcare facility within the previous 4 weeks and classified as indeterminable, if the patient had been discharged from a healthcare facility within the previous 4–12 weeks. A case of C. *difficile* infection in a patient, who had not been discharged from a healthcare facility (including hospital, nursing home, or other health care facilities) in the previous 12 weeks, was classified as community-acquired (CA-CDI) [16].

Associations were tested for significance by Pearson’s qui-squared test or Fisher’s exact test at a significance level of 5 %. Data entry and analyses were performed using Epi Data software (Epi-Info 3.3.2 [Centers for Disease Control and Prevention] for Windows [Microsoft Corp, Redmond, WA]) and STATA 10.5 (STATA Corp, College Station, TX).

## Results

From July until October 2012, 171 hospitalized patients with stool specimen positive for toxigenic *C. difficile* were included in our survey. The study patients consisted of 73 patients from which *C. difficile* toxin positive stool specimens, and of 98 patients from which *C. difficile* isolates were provided.

Of the 159 patients with known age, 127 (74.2 %) were 65 years or older, the median age was 76 years (range: 9–97 years), and the male to female ratio 2.2. Out of the 171 study patients, 162 (94.7 %) had diarrhea (Bristol Stool Chart types 5–7), 3 patients had exclusively abdominal cramps or vomiting, and 5 patients presented without any clinical signs compatible with a case of *C. difficile* infection. A total of 4.7 % (8/171), 1.2 % (2/171), and 8.8 (15/171) respectively, developed a severe disease in terms of requiring intensive care, surgical intervention, and fatal outcome, respectively. In 73.1 % (*n*  = 125) of the 171 study patients, CDI was likely acquired in the health care setting and in 19.9 % in the community; for 12 patients, the location of CD acquisition was indeterminable. For all 171 patients, isolates were available for ribotyping. A total of 46 different ribotypes were identified, of which PCR-ribotype (RT) 027 (20 %), 014 (15.8 %), 053 (10.5 %), 078 (5.3 %), and RT 002 (4.7 %) were the five most prevalent, accounting for 96 of the 171 (56 %) isolates. The PCR-ribotypes 027 and 053 were found only in patients from hospitals situated in East-Austria, comprised of Vienna, Burgenland, and Lower Austria; RT 014 was found in patients across all nine Austrian provinces and RT 078 was identified in patients from hospitals situated in Vienna and in five other provinces situated in South-, North-, and West-Austria (Table 2).

**Table 2:** The five most frequent PCR ribotypes of toxigenic *C. difficile* from 171 hospital patients by province of participating hospitals

	Austria	ProvincesNumber of *C. difficile* isolates								
		East-Austria			South-Austria		North-Austria		West-Austria	
		Vienna	Burgenland	Lower Austria	Carinthia	Styria	Upper Austria	Salzburg	Tyrol	Vorarlberg
Ribotypes	*N* = 171*n* (%)	*n*_1_ = 92*n* (%)	*n*_2_ = 8*n* (%)	*n*_3_ = 9*n* (%)	*n*_4_ = 11*n* (%)	*n*_5_ = 10*n* (%)	*n*_6_ = 13*n* (%)	*n*_7_ = 10*n* (%)	*n*_8_ = 12*n* (%)	*n*_9_ = 6*n* (%)
027	34 (19.9)	27 (29.3)	4 (50.0)	3 (33.3)	0 (0)	0 (0)	0 (0)	0 (0)	0 (0)	0 (0)
014	27 (15.8)	15 (16.3)	1 (12.5)	1 (11.1)	3 (27.3)	1 (10.0)	3 (23.1)	1 (10.0)	1 (8.3)	1 (16.7)
053	18 (10.5)	15 (16.3)	2 (25.0)	1 (11.1)	0 (0)	0 (0)	0 (0)	0 (0)	0 (0)	0 (0)
078	9 (5.3)	4 (4.3)	0 (0)	0 (0)	1 (9.1)	1 (10.0)	1 (7.7)	0 (0)	1 (8.3)	1 (16.7)
002	8 (4.7)	2 (2.2)	0 (0)	0 (0)	2 (18.2)	0 (0)	0 (0)	3 (30.0)	1 (8.3)	0 (0)
Other^a^	75 (43.9)	29 (31.5)	1 (12.5)	4 (33.3)	5 (45.5)	8 (80.0)	9 (69.2)	6 (60.0)	9 (75.0)	4 (66.7)

^a^003, 005, 006, 010, 012, 017, 018, 019, 020, 029, 046, 049, 056, 070, 081, 126, 131, 220, 251, 403, 404, 419, 448, 538, 574, 642, 644, 645, 646, 650, 651, 652, AI-12, AI-21, AI-3, AI-72, AI-75, AI-8, AI-82, AI-83, AI-9

Study patients 65 years and older were 3.81 times more frequently positive for RT053 as compared to study patients less than 65, at borderline statistical significance (95 %CI: 0.91, 15.96; *p*  = 0.06). No other dominate ribotype was found to be significantly associated with age.

Table 3 displays results of the in vitro antimicrobial susceptibility testing of the *C. difficile* isolates, segregated into the five most frequent ribotypes found among the study participants. All isolates were susceptible to metronidazole and vancomycin. *C. difficile* RT 002 isolates were 2.4 (95 % CI: 1.31, 4.29; *p* = 0.04) and RT 014 1.8 (PR: 1.78; 95 % CI: 1.07, 2.95, *p* = 0.0391) times more frequently found to be susceptible to all five antibiotics tested (“resistotype 0”), as compared each to all other identified ribotypes. All RT 027 (*n*  = 27) and RT 053 isolates (*n*  = 18) showed resistance against moxifloxacin. RT 027 displayed almost five times more frequently (95 %CI: 2.22, 10.93; *p*  < 0.001) monoresistance to moxifloxacin (resistotype 2), almost three times more frequently (95 % CI: 1.74, 4.94; *p*  < 0.001) clindamycin/moxifloxacin resistance (resistotype 4), and 12 times more frequently (95 % CI: 1.30, 112.62; *p*  = 0.0252) moxifloxacin/rifampicin resistance (resistotype 6), as compared to all other ribotypes. PCR-RT 053 exhibited clindamycin/moxifloxacin resistance almost four times more frequently (95 %CI: 2.43, 6.33; *p*  = 0.000), as compared to the other ribotypes, and the tri-resistance against clindamycin/moxifloxacin/rifampicin (resistotype 7) 5.3 times more frequently than all other ribotypes (95 % CI: 1.94, 14.51). RT 078 was not found to be associated with any of the seven resistotypes tested. Ribotype 053 was the single ribotype found to be associated with severe CDI (PR: 3.04; 95 %CI: 1.24, 7.44; *p*  = 0.012) (Table 4), also after adjustment for age.

**Table 3:** Results of the *in vitro* antimicrobial susceptibility testing of *Clostridium difficile* isolates according to European Committee on Antimicrobial Susceptibility Testing (EUCAST) minimal inhibitory concentration (MIC) and Clinical and Laboratory Standards Institute (CLSI) MIC breakpoints, respectively distributed by the five most frequent ribotypes among the study participants (*N*  = 96)

	Metronidazole *n* (%)			Vancomycin *n* (%)			Clindamycin *n* (%)			Moxifloxacin *n* (%)			Rifampicin *n* (%)		
Ribotypes	S (≤ 2 µg/ml)	I	R	S (≤ 2 µg/ml)	I	R	S (≤ 2 µg/ml)	I (4 µg/ml)	R (≥ 8 µg/ml)	S (≤ 2 µg/ml)	I (4 µg/ml)	R (≥ 8 µg/ml)	S (≤ 0.006 µg/ml)	I	R (≥ 32 g/ml)
027 (*N*1 = 34)	34 (100)	0	0	34 (100)	0	0	14 (41.2)	9 (26.5)	11 (32.4)	0 (0)	0	34 (100)	27 (79.4)	0	7 (20.6)
014 (*N*2 = 27)	27 (100)	0	0	27 (100)	0	0	16 (59.3)	7 (25.9)	4 (14.8)	22 (81.5)	1 (3.7)	4 (14.8)	26 (96.3)	0	1 (3.7)
053 (*N*3 = 18)	18 (100)	0	0	18 (100)	0	0	1 (5.6)	1 (5.6)	16 (88.9)	0	0	18 (100)	13 (72.2)	0	5 (27.8)
078 (*N*4 = 9)	9 (100)	0	0	9 (100)	0	0	3 (33.3)	4 (44.4)	2 (22.2)	5 (55.6)	0	4 (44.4)	8 (88.9)	0	1 (11.1)
002 (*N*5 = 8)	8 (100)	0	0	8 (100)	0	0	5 (62.5)	2 (25.0)	1 (12.5)	8 (100)	0	0	8 (100)	0	0
*P-value*									*0.000* ^a^			*0.000* ^a^			*0.10* ^a^

^a^Fisher’s exact test

**Table 4:** Epidemiological classification of the *Clostridium difficile* infection (CDI) cases and cases of severe CDI by the five most frequent ribotypes and others found among the study participants (N_total_=171)

	Epidemiological classification			Criteria for severe CDI			
Ribotypes	HA*n* (%) (95 %CI)	CA*n* (%)	Indeterminable*n* (%)	Surgical intervention*n* (%)	Intensive care *n* (%)	30-day case fatality *n* (%)	Total severe CDI *n* (%)
027 (*N*_1_ = 34; 19.9 %)	29 (85.3) (70–94 %)	3 (8.8)	2 (5.9)	0 (0)	1 (2.9)	3 (8.8)	3 (8.8)
014 (*N*_2_ = 27; 15.8 %)	22 (81.5) (64–93 %)	4 (14.8)	1 (3.7)	1 (3.7)	2 (7.4)	3 (11.1)	4 (14.8)
053 (*N*_3_ = 18; 10.5 %)	13 (72.2) (49–89 %)	3 (16.7)	2 (11.1)	*1 (5.6)*	*1 (5.6)*	*4 (22.2)*	*5 (27.8)*
078 (*N*_4_ = 9; 5.3 %)	7 (77.8) (44–96 %)	2 (22.2)	0 (0)	0 (0)	1 (11.1)	0 (0)	1 (11.1)
002 (*N*_5_ = 8; 4.7 %)	5 (62.5) (28–89 %)	2 (25.0)	1 (12.5)	0 (0)	0 (0)	1 (12.5)	1 (12.5)
Other (*N*_6_ = 75)	49 (65.3) (54–75 %)	20 (26.7)	6 (8.0)	0 (0)	3(0)	4 (5.3)	5 (6.6)
*TOTAL (N*_*total*_= *171)*	*125 (73.1)*	*34 (19.9)*	*12 (7.0)*	*2 (1.2)*	*8 (1.2)*	*15 (8.8)*	*19 (11.1)*

All of the five most prevalent ribotypes were found to be more frequently acquired in the health care setting than in the community.

## Discussion

The global emergence of CDI in the past decade followed highly-publicized *C. difficile* outbreaks in the USA and Canada that were associated with increased rates of disease recurrence and mortality [17–19]. The outbreaks were caused by a previously uncommon, fluoroquinolone resistant variant of *C. difficile* genotyped as ribotype 027. A European hospital-based study [1] together with a US analysis of *C. difficile* ribotype data by Walk et al. [20] indicated that the most frequent ribotypes in the industrialized world are 014, 020, 027, and 078.

We found 027 to be the most prevalent ribotype, accounting for 20 % of all isolates from the 171 hospitalized, *C. difficile* positive patients. The second most prevalent ribotype was 014, followed by RT 053, RT 078, and RT 002. In Austria, the first case of *C. difficile* RT 027 was identified in a 69-year-old British woman admitted to a local hospital in the Austrian western province Tyrol in 2006 [21]. In April 2008, *C. difficile* RT 027 infections were first identified in Austrian citizens, one hospitalized in Styria, a southern province, and one in Vienna [22]. Since August 2006, the *C. difficile* Reference Laboratory ribotyped approximately 2700 human *C. difficile* isolates, received from all nine Austrian provinces, without identifying any further *C. difficile* RT 027 isolates until 2008. In 2008, a steep increase in the number of cases of RT 027 infections was observed, all originating from four Viennese hospitals. From November 2008 to mid-April 2009, 36 patients with *C. difficile* RT 027 infection were identified at the National Reference Laboratory [23]. Although the outbreak apparently ceased, our current findings 4 years later demonstrate the remaining dominance of RT 027 in Vienna (34 of 92 CDI-cases from the 10 Viennese hospitals). In addition to Vienna, the surrounding province Lower Austria (3 of 9 CDI-cases) and the neighboring province Burgenland (4 of 8 CDI-cases) were also affected by RT 027, in contrast to the remaining six other federal provinces. The observed dominance of RT 027 in patients from hospitals in Eastern Austria could be disputed as due to a selection bias in view of the over-representativeness of the hospitals in the eastern province Vienna (accounting for 10 of the 18 participating hospitals). To account for the over-representativeness of Vienna hospitals, as well as for the different province population densities, we can consider a quarter of the 27 cases of RT 027-infection in patients from Viennese hospitals as representative. Together with the seven cases of RT 027 infection in patients from the two other eastern provinces (i.e., Burgenland, Lower Austria), these findings demonstrate a dominance of RT 027 in East-Austria, as compared to the other six Austrian provinces (no RT 027 case observed).

*C. difficile* is an important nosocomial pathogen, as illustrated by our findings concerning 73 % of the 171 CDI-cases acquired in the healthcare setting. The five dominant ribotypes 027, 014, 053, 078, and 002 were similarly distributed among the health care-associated cases. No single ribotype was found to be associated with community acquisition. Our findings support the previous conclusion of Indra et al. [11], that *C. difficile* is still a pathogen of the health care facility setting. However, the total portion of community-acquired cases among the 171 CDI-cases was surprisingly high, at almost 20 %. In November 2008, among three Austrian hospitals participating in a European study, only 8 % of the CDI cases documented were found to be community acquired [1]. Among CDI cases identified in the USA within the Emerging Infections Program data in 2010, 52 % were already present at hospital admission, although they were largely health care facility related by means of outpatients and nursing home residents. The Centers for Disease Control and Prevention concluded that nearly all CDIs are related to various health care settings where predisposing antibiotics are prescribed and *C. difficile* transmission occurs [24].

PCR ribotype 078 has been detected frequently in farming animals, in retail meat products and was associated mainly with the community setting, indicating also a zoonotic transmission [8, 9, 25]. In our survey, RT 078 accounted for 5 % of all isolates and was found in Vienna most (4 cases), and with one case each in five other federal provinces in South, North, and West Austria, more rural areas. Only two of the nine RT 078 cases were classified as community acquired. Information on exposure to farm animals or on patients’ diet was not available. However, the over-representativeness of urban hospitals among the participating hospitals of our survey does not allow reliable conclusion on the origin of this particular ribotype in Austria.

A single ribotype—the RT 053—was identified to be significantly associated with severe CDI, including need for intensive care, surgical treatment, or fatal outcome. While this association was still significant after adjustment for age, no data on comorbidities were available to control for possible confounding of the observed association. The so-called hypervirulent ribotypes 027 and 078 did not show an association with severe CDI or fatal outcome in our study. Our small study sample size might hinder any strong conclusions drawn from our findings, but previous publications have also reported lack of strong evidence for an association between the so-called hypervirulent ribotypes or other strain characteristics (such as mutation/deletions in *tcdC* or binary toxin production) and pathogen virulence [20, 26]. Therefore, use of resource-consuming genotyping in guiding treatment or infection control measures must be critically discussed. Barbut and Rupnik [27] stated that the most important control measure to be implemented is CDI surveillance and more timely response to a case, regardless of whether it is caused by a known or a newly emerging potential hyper-virulent genotype. Other experts recommend that ribotyping should be undertaken on all samples to detect not only outbreaks due to epidemic strains, but also outbreaks with non-epidemic strains, feasible due to poor environmental disinfection or other poor hygiene practices [28].

The steep increase in the role of RT 027 in Austria since 2008 is in sharp contrast to the trend in the UK, where the incidence of the “*more virulent epidemic ribotype 027 (one of the key reasons leading to the development of an enhanced surveillance program) has declined markedly in the few years since the inception*” of the Health Protection Agency’s *Clostridium difficile* Ribotyping Network [28]. All 34 RT 027 isolates were shown to exhibit *in vitro* resistance against moxifloxacin. He et al. [29] sequenced the genomes of a global collection of 151 *C. difficile* 027 strains and found that the separate acquisitions of fluoroquinolone resistance and a conjugative transposon (Tn*6192*) in two distinct lineages of *C. difficile* 027 (FQR1and FQR2) were the key genetic changes linked to its rapid emergence during the early 2000s. The presence of Tn*6192* is the only other significant genetic trait, aside from the fluoroquinolone resistance single nucleotide polymorphism, which differs from the pre-epidemic isolates; whether the genes carried by this element have any phenotypic effect on the core genome is unknown. Also all 18  *C. difficile* 053 isolates exhibited resistance against moxifloxacin in our survey. Similar findings were reported by Tenover et al. [30], studying the resistance profiles from *C. difficile* clinical isolates from patients in North America: moxifloxacin resistance was present in > 90 % of PCR-ribotype 027 and 053 isolates but was less common among other ribotypes. In RT 053 isolates, clindamycin/moxifloxacin/rifampin resistance was five times more frequently exhibited, as compared with all other ribotypes. Fluoroquinolones are still one of the most commonly prescribed antibiotic classes in Austria. Hence, the selective pressure for the acquisition and maintenance of fluoroquinolone resistance in the healthcare setting could further favor the spread of the fluoroquinolone resistant *C. difficile* clones. Elderly patients are more likely to acquire *C. difficile* infection in general and in particular, infections with resistant pathogens [31]. In our study cohort, 74 % of the CDI-patients were 65 years or older. RT 053, of which 17 of the 18 isolates exhibited at least a bi-resistance, was found to be associated at borderline statistical significance with older age.

Nosocomial diarrhea is no longer a mere nuisance but a serious public health problem [2]. In 2013, the Wilhelminenspital (Vienna, Austria), a large tertiary care community hospital with 1081 beds and 357,892 patient days, was affected by a CDI-outbreak [32]. While their CDI-numbers were stable at < 200 patients per year from 2009 to 2011 (0.56, 0.51, and 0.50 per 1,000 patient days, respectively), an increase to 313 patients was observed in 2012 (0.88/1000 patient days). In the first 5 months of 2013, a further increase in CDI patients was detected (*n*  = 294; 1.98/1000 patient days). Severe disease was recorded for 31 % of the patients, and in 131 (25 %) of the cases, the patient died within 30 days of diagnosis. Of the cases in which the patient died, 57 (43.4 %) involved ribotype 027. Implementation of an antibiotic stewardship program including formulary restriction and direct feedback to the prescriber resulted in a 46 % decrease in average number of CDI-cases per month. Moxifloxacin use was reduced from 1038 ± 109 defined daily doses (DDD) per month (January to May, period 1) to 42 ± 10 DDD per month (June to December, period 2) (*P*  = 0.0045). Because use of any antimicrobial has the potential to induce the manifestation of CDI, antimicrobial stewardship programs that promote judicious use of antimicrobials, and environmental and infection control-related efforts must be encouraged [33, 34]. We hope that our study can contribute to raise awareness and to support good medical practice in the fight against this often underestimated illness.

### Conflicts of interest

The authors declare that there a no actual or potential conflicts of interest related to this article.

## References

[1] Bauer MP, Notermans DW, van Benthem BH (2011). *Clostridium difficile* infection in Europe: a hospital-based survey. Lancet.

[2] Allerberger F (2012). Time to act against *Clostridium difficile* infection. Clin Microbiol Infect.

[3] Warny M, Pepin J, Fang A (2005). Toxin production by an emerging strain of *Clostridium difficile* associated with outbreaks of severe disease in North America and Europe. Lancet.

[4] Health Protection Agency. Voluntary surveillance of Clostridium difficile, England, Wales and Northern Ireland: 2013. Infection report 8 (Number 7) Published electronically on: 21 February 2014. https://www.gov.uk/government/uploads/system/uploads/attachment_data/file/339374/Clostridium_difficile_voluntary_surveillance_2013.pdf. Accessed: 25 May 2015.

[5] Goorhuis A, Van der Kooi T, Vaessen N (2007). Spread and epidemiology of *Clostridium difficile* polymerase chain reaction ribotype 027/toxinotype III in The Netherlands. Clin Infect Dis.

[6] Kuijper EJ, Barbut F, Brazier JS (2008). Update of *Clostridium difficile* infection due to PCR ribotype 027 in Europe. Euro Surveill.

[7] Goorhuis A, Bakker D, Corver J (2008). Emergence of *Clostridium difficile* infection due to a new hypervirulent strain, polymerase chain reaction ribotype 078. Clin Infect Dis.

[8] Hensgens MP, Keessen EC, Squire MM (2012). *Clostridium difficile* infection in the community: a zoonotic disease?. Clin Microbiol Infect.

[9] Songer JG, Trinh HT, Killgore GE, Thompson AD, McDonald LC, Limbago BM (2009). *Clostridium difficile* in retail meat products, USA 2007. Emerg Infect Dis.

[10] Barbut F, Jones G, Eckert C (2011). Epidemiology and control of *Clostridium difficile* infections in healthcare settings: an update. Curr Opin Infect Dis.

[11] Indra A, Lassnig H, Baliko N (2009). *Clostridium difficile*: a new zoonotic agent?. Wien Klin Wschr.

[12] Gilca R, Frenette C, Theriault N, Fortin E, Villeneuve J (2012). Attributing cause of death for patients with *Clostridium difficile* infection. Emerg Infect Dis.

[13] Hell M, Sickau K, Chmelizek G (2012). Absence of *Clostridium difficile* in asymptomatic hospital staff. AJIC.

[14] Indra A, Huhulescu S, Schneeweis M (2008). Characterization of *Clostridium difficile* isolates using capillary gel electrophoresis-based PCR ribotyping. J Med Microbiol.

[15] Huhulescu S, Sagel U, Fiedler A (2011). Rifaximin disc diffusion test for in vitro susceptibility testing of *Clostridium Difficile*. J Med Microbiol.

[16] Cohen SH1, Gerding DN, Johnson S, Kelly CP (2010). Society for healthcare epidemiology of America; infectious diseases society of America. Clinical practice guidelines for *Clostridium difficile* infection in adults: 2010 update by the society for healthcare epidemiology of America (SHEA) and the infectious diseases society of America (IDSA). Infect Control Hosp Epidemiol.

[17] McDonald LC, Killgore GE, Thompson A (2005). An epidemic, toxin gene-variant strain of *Clostridium Difficile*. N Engl J Med.

[18] Muto CA, Pokrywka M, Shutt K (2005). A large outbreak of *Clostridium difficile*-associated disease with an unexpected proportion of deaths and colectomies at a teaching hospital following increased fluoroquinolone use. Infect Control Hosp Epidemiol.

[19] Pepin J, Valiquette L, Cossette B (2005). Mortality attributable to nosocomial *Clostridium difficile*-associated disease during an epidemic caused by a hypervirulent strain in Quebec. CMAJ.

[20] Walk ST, Micic D, Jain R (2012). *Clostridium difficile* ribotype does not predict severe infection. Clin Infect Dis.

[21] Indra A, Huhulescu S, Hasenberger P (2006). First isolation of *Clostridium difficile* PCR ribotype 027 in Austria. Euro Surveill.

[22] Indra A, Huhulescu S, Kernbichler S (2008). First cases of *Clostridium difficile* PCR ribotype 027 acquired in Austria. Euro Surveill.

[23] Indra A, Huhulescu S, Fiedler A, Kernbichler S, Blaschitz M, Allerberger F (2009). Outbreak of *Clostridium difficile* 027 infection in Vienna, Austria 2008–2009. Euro Surveill.

[24] Centers for Disease Control and Prevention (2012). Vital signs: preventing *Clostridium difficile* infections. MMWR Morb Mortal Wkly Rep.

[25] Rupnik M, Widmer A, Zimmermann O, Eckert C, Barbut F (2008). *Clostridium difficile* toxinotype V, ribotype 078, in animals and humans. J Clin Microbiol.

[26] Sirard S, Valiquette L, Fortier LC (2011). Lack of association between clinical outcome of *Clostridium difficile* infections, strain type, and virulence-associated phenotypes. J Clin Microbiol.

[27] Barbut F, Rupnik M (2012). 027, 078, and others: Going beyond the numbers (and away from the hypervirulence). Clin Infect Dis.

[28] Patel TA, Smith R, Hopkins S (2013). Ribotyping in the detection of *Clostridium difficile* outbreaks in a single university hospital. J Hosp Infect.

[29] He M, Miyajima F, Roberts P (2013). Emergence and global spread of epidemic healthcare-associated *Clostridium Difficile*. Nat Genet.

[30] Tenover FC, Tickler E, Persing D (2012). Antimicrobial-resistant strains of *Clostridium difficile* from North America. Antimicrob Agents Chemother.

[31] Owens RC, Donskey CJ, Gaynes RP, Loo VG, Muto CA (2008). Antimicrobial-associated risk factors for *Clostridium difficile* Infection. Clin Infect Dis.

[32] Wenisch JM, Equiluz-Bruck S, Fudel M (2014). Decreasing *Clostridium difficile* infections by an antimicrobial stewardship program that reduces moxifloxacin use. Antimicrob Agents Chemother.

[33] de With K, Allerberger F, Amann S, et al. S3-Leitlinie Strategien zur Sicherung rationaler Antibiotika-Anwendung im Krankenhaus. AWMF-Registernummer 092/001. 2013. http://www.awmf.org/uploads/tx_szleitlinien/092-001l_S3_Antibiotika_Anwendung_im_Krankenhaus_2013-12.pdf. Accessed: 9 March 2015.

[34] Davies KA, Longshaw CM, Davis GL (2015). Underdiagnosis of *Clostridium difficile* across Europe: the European, multicentre, prospective, biannual, point-prevalence study of *Clostridium difficile* infection in hospitalised patients with diarrhoea (EUCLID). Lancet Infect Dis.

